# An asymmetric fission island driven by shell effects in light fragments

**DOI:** 10.1038/s41586-025-08882-7

**Published:** 2025-04-30

**Authors:** P. Morfouace, J. Taieb, A. Chatillon, L. Audouin, G. Blanchon, R. N. Bernard, N. Dubray, N. Pillet, D. Regnier, H. Alvarez-Pol, F. Amjad, P. André, G. Authelet, L. Atar, T. Aumann, J. Benlliure, K. Boretzky, L. Bott, T. Brecelj, C. Caesar, P. Carpentier, E. Casarejos, J. Cederkäll, A. Corsi, D. Cortina-Gil, A. Cvetinović, E. De Filippo, T. Dickel, M. Feijoo, L. M. Fonseca, D. Galaviz, G. García-Jiménez, I. Gasparic, E. I. Geraci, R. Gernhäuser, B. Gnoffo, K. Göbel, A. Graña-González, E. Haettner, A.-L. Hartig, M. Heil, A. Heinz, T. Hensel, M. Holl, C. Hornung, A. Horvat, A. Jedele, D. Jelavic Malenica, T. Jenegger, L. Ji, H. T. Johansson, B. Jonson, B. Jurado, N. Kalantar-Nayestanaki, E. Kazantseva, A. Kelic-Heil, O. A. Kiselev, P. Klenze, R. Knöbel, D. Körper, D. Kostyleva, T. Kröll, N. Kuzminchuk, B. Laurent, I. Lihtar, Yu. A. Litvinov, B. Löher, N. S. Martorana, B. Mauss, S. Murillo Morales, D. Mücher, I. Mukha, E. Nacher, A. Obertelli, E. V. Pagano, V. Panin, J. Park, S. Paschalis, M. Petri, S. Pietri, S. Pirrone, G. Politi, L. Ponnath, A. Revel, H.-B. Rhee, J. L. Rodríguez-Sánchez, L. Rose, D. Rossi, P. Roy, P. Russotto, C. Scheidenberger, H. Scheit, H. Simon, S. Storck-Dutine, A. Stott, Y. L. Sun, C. Sürder, Y. K. Tanaka, R. Taniuchi, O. Tengblad, I. Tisma, H. T. Törnqvist, M. Trimarchi, S. Velardita, J. Vesic, B. Voss, F. Wamers, H. Weick, F. Wienholtz, J. Zhao, M. Zhukov

**Affiliations:** 1 CEA, DAM, DIF, Arpajon, France; 2 Université Paris-Saclay, CEA, Laboratoire Matière en Conditions Extrêmes, Bruyères-le-Châtel, France; 3 Université Paris-Saclay, CNRS, IJC Lab, Orsay, France; 4 CEA, DES, IRESNE, DER, SPRC, Cadarache, Saint-Paul-Lès-Durance, France; 5 IGFAE, University of Santiago de Compostela, Santiago de Compostela, Spain; 6 GSI Helmholtzzentrum für Schwerionenforschung, Darmstadt, Germany; 7 CEA Saclay, IRFU/DPhN, Centre de Saclay, Gif-sur-Yvette, France; 8 Technische Universität Darmstadt, Fachbereich Physik, Institut für Kernphysik, Darmstadt, Germany; 9 Helmholtz Research Academy Hesse for FAIR (HFHF), GSI Helmholtzzentrum für Schwerionenforschung, Darmstadt, Germany; 10 Instituto de Física Corpuscular, CSIC - Univ. of Valencia, Valencia, Spain; 11 Goethe-Universität Frankfurt, Frankfurt am Main, Germany; 12 Jozef Stefan Institute, Ljubljana, Slovenia; 13 CINTECX, Universidade de Vigo, DSN, Department of Mechanical Engineering, Vigo, Spain; 14 Department of Physics, Lund University, Lund, Sweden; 15 INFN Sezione di Catania, Catania, Italy; 16 II. Physikalisches Institut, Justus-Liebig-Universität, Giessen, Germany; 17 Instituto de Física, Universidade Federal Fluminense, Niterói, Brazil; 18 Laboratório de Instrumentação e Física Experimental de Partículas, LIP, Lisbon, Portugal; 19 Physics Department, Faculty of Sciences, University of Lisbon, Lisbon, Portugal; 20 RBI Zagreb, Zagreb, Croatia; 21 Dipartimento di Fisica e Astronomia ‘Ettore Majorana’, Università degli Studi di Catania, Catania, Italy; 22 Technische Universität München, Garching, Germany; 23 Institutionen för Fysik, Chalmers Tekniska Högskola, Gothenburg, Sweden; 24 Helmholtz-Zentrum Dresden-Rossendorf, Institute of Radiation Physics, Dresden, Germany; 25 Université de Bordeaux, CNRS, LP2IB, Gradignan, France; 26 ESRIG, University of Groningen, Groningen, The Netherlands; 27 School of Physics, Engineering and Technology, University of York, York, UK; 28 University of Guelph, Guelph, Ontario Canada; 29 Institut für Kernphysik der Universität zu Köln, Cologne, Germany; 30 INFN Laboratori Nazionali del Sud, Catania, Italy; 31 Institute for Basic Science, Center for Exotic Nuclear Studies, Daejeon, Republic of Korea; 32 CITENI, Campus Industrial de Ferrol, Universidade de Coruña, Ferrol, Spain; 33 Instituto de Estructura de la Materia, CSIC, Madrid, Spain; 34 Università degli Studi di Messina, Messina, Italy

**Keywords:** Experimental nuclear physics, Nuclear fusion and fission, Nuclear astrophysics

## Abstract

Nuclear fission leads to the splitting of a nucleus into two fragments^1,2^. Studying the distribution of the masses and charges of the fragments is essential for establishing the fission mechanisms and refining the theoretical models^3,4^. It has value for our understanding of r-process nucleosynthesis^5,6^, in which the fission of nuclei with extreme neutron-to-proton ratios is pivotal for determining astrophysical abundances and understanding the origin of the elements^7^ and for energy applications^8,9^. Although the asymmetric distribution of fragments is well understood for actinides (elements in the periodic table with atomic numbers from 89 to 103) based on shell effects^10^, symmetric fission governs the scission process for lighter elements. However, unexpected asymmetric splits have been observed in neutron-deficient exotic nuclei^11^, prompting extensive further investigations. Here we present measurements of the charge distributions of fission fragments for 100 exotic fissioning systems, 75 of which have never been measured, and establish a connection between the neutron-deficient sub-lead region and the well-understood actinide region. These new data comprehensively map the asymmetric fission island and provide clear evidence for the role played by the deformed *Z* = 36 proton shell of the light fragment in the fission of sub-lead nuclei. Our dataset will help constrain the fission models used to estimate the fission properties of nuclei with extreme neutron-to-proton ratios for which experimental data are unavailable.

## Abstract (web summary)

Measurements of fission fragments for 100 fissioning systems are used to map an asymmetric fission island, providing evidence for the role played by the deformation induced by a closed 36-proton shell.

## Main

Nuclear fission, a process during which a nucleus undergoes extreme deformations before splitting into two energy-releasing fragments, has traditionally been compared to the division of a liquid drop, which emphasizes the dynamic evolution of the compound nucleus. This view, however, has expanded to include microscopic structural effects due to quantum behaviour. These effects are essential for the outcome of fission. Despite being discovered over 85 years ago^1,2^, the intricate interplay between the macroscopic and microscopic effects presents a significant theoretical challenge that remains to be solved^4^.

The significance of understanding fission fragment distributions extends beyond pure scientific curiosity, as they impact terrestrial energy production, reactor safety and various astrophysical phenomena. Knowing fission fragment mass and charge distributions is important, particularly for assessing criticality in nuclear reactors, as certain fission fragments can capture neutrons and disrupt the nuclear chain reaction. Additionally, understanding these distributions is important for evaluating the residual heat after a reactor shutdown^8,9^. Furthermore, fission plays a pivotal role in the survival probability of super-heavy elements^12^, in determining the antineutrino flux in nuclear reactors through the β decay of neutron-rich fission fragments^13^ and in the recycling path of r-process nucleosynthesis, a key process that generated a large fraction of all the heavy elements we have observed^5,6^. However, the experimental data currently available are limited to a narrow range of the neutron-to-proton ratio (*N*/*Z* ≈ 1.5) and are insufficient for the needs of astrophysical modelling, which requires information on more exotic nuclei (*N*/*Z* ≈ 1.8), where experimental data are sparse. This gap necessitates a reliance on theoretical models for predicting fission fragment distributions^7^, which underscores the importance of expanding experimental data to refine these models.

In-depth studies of the uranium and plutonium region have revealed mass-asymmetry and specific charge distribution preferences^14^, which are attributed to both deformed spherical and octupole shells stabilizing the position of the heavy-fragment distribution^3^. Other experiments revealed a shift from asymmetric to symmetric fission in neutron-deficient thorium isotopes, including the discovery of a new compact symmetric fission mode^15^. Evidence also indicates that there are asymmetric contributions in the fission of pre-actinides near the stability line^16^. Previous research has indicated that the fission of lighter nuclei would primarily exhibit symmetry, as dictated by the liquid drop model. However, this paradigm shifted significantly following the experimental revelation of a markedly asymmetric mass division in the exotic ^180^Hg nucleus^11^. This unforeseen behaviour has spurred extensive theoretical and experimental investigations.

In experimental studies, two primary approaches have been employed to explore this unique region. The first method involves β-delayed fission^11,17,18^. This technique is applicable to nuclei for which *Q*_β_, the total energy released during a β-decay process, is close to or slightly lower than the fission barrier *B*_f_, limiting the number of measurable nuclei to a few. The alternative technique is the fusion–fission reaction, which uses a specific combination of beam and target to access the neutron-deficient region^19–27^. In ref. ^27^, the charge of the fragment was measured exclusively for the light fragment using a variable-mode high-acceptance spectrometer. That experiment, which took advantage of fusion–fission reactions, is the only one to have partially measured the nuclear charge of fragments. Other experiments using the same reaction mechanism have typically extracted fragment masses or mass ratios along with the kinetic energy using detectors such as multichannel plate or multi-wire proportional chambers. The mass resolution achieved with this approach ranges from 3 to 6 mass units, r.m.s., according to the specific features of the experiment. Moreover, the fissioning system is populated at relatively high excitation energies (20 to 50 MeV usually) and angular momenta (tens of *ħ*), thereby reducing significantly the influence of nuclear structure. In ref. ^28^, this wealth of mass distributions was used to infer the mean nuclear charge in the heavy and light peaks. For many of the experiments compiled in that paper, the statistics appear to be limited (tens to hundreds of events). Moreover, to extract information related to the nuclear charge from the measured mass distribution, the authors of ref. ^28^ assumed that the unchanged charge distribution approximation was valid in this context. Through this indirect method, these authors pointed out the dominant role of a proton-deformed shell for the light fragments, particularly around *Z* = 36.

On the theoretical side, a surge of studies has resulted in a variety of approaches and interpretations. Combined macroscopic and microscopic models^29,30^ indicate that there is no direct correlation between fragment shell structure and asymmetric splitting in this area, and they have predicted an island of asymmetric fission^31^. Conversely, scission point models emphasize the significance of shell effects in determining the final fragments^32–34^. Microscopic models using nuclear density functional theory with the Skyrme or Gogny phenomenological effective nucleon–nucleon interactions anticipate a shift towards asymmetric fission in the neutron-deficient region, which is relatively unaffected by excitation energy^35,36^. Last, recent microscopic mean-field calculations^3,37^ underscore the role of octupole correlations as a universal explanation for the observed fission patterns.

The sub-lead region, accessible experimentally and distinguished by its low *N*/*Z* ratio of 1.25 approaching the proton drip line, provides a distinct and unique testing ground for scrutinizing the isospin dependence (the proton–neutron symmetry) of fission models. In this study, we unveil fission fragment charge distributions of 100 fissioning systems and provide direct experimental information regarding fragment charges compared to what has been done in previous studies^28^. These systems were propelled to relativistic energies at the accelerator facility of GSI in Darmstadt, Germany, and underwent excitation through electromagnetic interactions upon traversing a lead target. The level of resolution achieved (*σ* ≈ 0.13 charge units) enabled us to elucidate the role of proton shells and the magnitude of odd–even staggering associated with pairing correlations in the fission process. Our investigation extends from the sub-lead region to the actinide region and charts the boundaries of a new island of asymmetric fission. In a single experiment, we systematically captured the entire charge distribution in this important neutron-deficient region, thus imposing robust constraints on fission models.

## Production and identification of radioactive beams

**Fig. 1:**
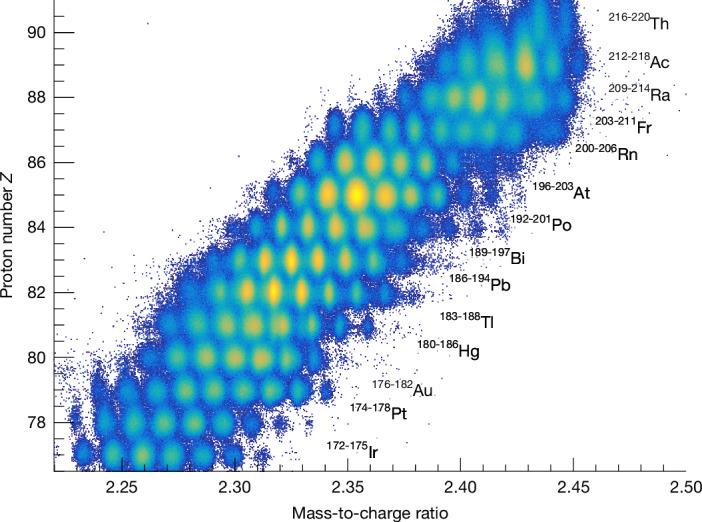
Particle identification plot of the secondary beam. Particle identification plot of the radioactive beams produced and selected by the FRS. The isotopes listed in black correspond to the ones studied through Coulomb excitation.

The experiment was performed at GSI, Germany, within the Reactions with Relativistic Radioactive Beams/Studies On Fission with GLAD (R^3^B-SOFIA) collaboration. The exotic secondary beams were produced by the fragmentation of a ^238^U primary beam accelerated to an energy of 1*A* GeV, corresponding to 87.6% of the speed of light, after impinging on a 1,625 mg cm^−^^2^ thick beryllium production target located at the entrance of the fragment separator (FRS)^38^. The secondary beams of interest, the fissioning systems, were then selected and transported through the FRS to the experimental cave (Cave C). The different incoming beams were identified event by event using the Δ*E*–*Bρ*–time of flight (ToF) method^39,40^. To map the whole region of interest covering 100 nuclei, from iridium to thorium, 12 different FRS settings were needed. The identification plot of all fissioning systems transmitted in the different FRS settings is shown in Fig. 1.

## Fission reaction and fragment identification

The incoming nuclei were induced to fission by Coulomb excitation at relativistic energies through the interaction of the beam with high-*Z* targets (Methods). In our experiment, two 1.5-mm-thick natural lead targets were mounted as the cathodes in an active target. The difference in the energy losses between the beam and fission fragments allowed us to determine in which target fission took place. In addition to the two lead cathodes, one 0.5-mm-thick low-*Z* cathode made of carbon was also part of the active target. Using this other target, we measured and, thus, could allow for the subtraction of fragmentation-fission reactions as described in ref. ^41^. The focus of the study was Coulomb-induced fission, which led to fission at low excitation energies, between the fission barrier and 10 MeV above it, where the structure and pairing effects were preserved^14^. Note that, although the structure and pairing effects were preserved, damping of the shell effect could occur because of the extra energy above the fission barrier. However, the extent of this quenching is not yet fully understood or quantified in the context of fission. With the given excitation energy distribution, second-chance fission can occur, making it impossible to distinguish from the data whether a neutron was emitted before fission. Nonetheless, the contribution of second-chance fission was modest, as quantified in Extended Data Tables 3 and 4 using the GEF 2023/1.2 code (General Description of Fission Observables)^42^. This small contribution had a minor effect on the charge distributions, as it evolved gradually across neighbouring masses.

After a fission reaction had occurred, both fragments were emitted at a forward angle within a narrow cone of 35 mrad in the laboratory frame. They were detected in coincidence in the R^3^B-SOFIA set-up. For a given fission event, the probability of properly identifying the charge of both fission fragments was about 90%. The resolution obtained (*σ* = 0.13) allowed us to unambiguously identify the fission fragment charges, extract the odd–even staggering in the population of the fragments due to the pairing interaction and investigate the role of proton-shell closures during the fission of the investigated isotopes. The final charge yield, normalized to 200%, was obtained systematically and consistently for all the fissioning nuclei listed in Extended Data Tables 3 and 4. Those tables also list the estimated average mean excitation energy above the fission barrier for all systems studied. The excitation energy was inferred using the GEF 2023/1.2 code^42^. We used this phenomenological Monte Carlo approach to calculate for each fission event the fragment properties at scission, which is the point in the nuclear fission process where the nucleus splits into two or more fragments. The properties calculated include the charge, mass, angular momentum and kinetic energy. The excitation energy spectrum from Coulomb excitation, used as an input in the GEF calculations, was evaluated for all nuclei with the empirical parametrization of the giant resonances^14,41^ using the ground-state deformation from the AMEDEE database^43^ and based on large-scale Hartree–Fock–Bogoliubov calculations using the Gogny D1S nucleon–nucleon interaction^44,45^. However, estimating multi-chance fission probabilities in this new region remains challenging due to the lack of precise data.

To provide a global view of the evolution of fission for the investigated fissioning systems, we define the asymmetry parameter:

1 $${\mathcal{A}}=1-\frac{Y({Z}_{{\rm{sym}}})+\frac{Y({Z}_{{\rm{sym}}}-1)+Y({Z}_{{\rm{sym}}}+1)}{2}}{Y({Z}_{\max })+\frac{Y({Z}_{\max }-1)+Y({Z}_{\max }+1)}{2}},$$

which corresponds to the ratio between the symmetric yield *Y*(*Z*_sym_) and the maximum yield *Y*(*Z*_max_) averaged over the neighbouring yields to compensate for the odd–even staggering in the elemental distributions, with *Z*_max_, the charge corresponding to the highest yield for a given fissioning system: *Y*(*Z*_max_) = max(*Y*(*Z*))

**Fig. 2:**
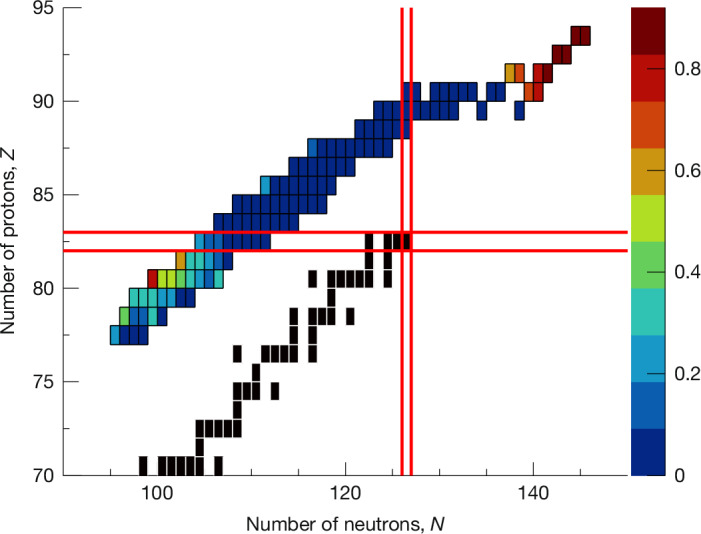
Map of the evolution of asymmetric fission. Experimental asymmetry as defined in equation (1). The data showing the asymmetric fission island in the actinide region are taken from previous experimental data with the SOFIA set-up (see text for details)^39,40^. All other nuclei are from the current dataset, and their charge yields are displayed in Extended Data Fig. 4. The black squares represent the valley of stability, and the red lines show the magic numbers *Z* = 82 and *N* = 126.

All measured charge yields are shown in Extended Data Fig. 4. A mapping of the asymmetry metric over the range of investigated nuclei is shown in Fig. 2. In addition to the current dataset, previous results from R^3^B-SOFIA experiments focused on selected ^237,238^Np, ^234,235^U, ^228,229,231,232^Pa and ^221,222,223,225,226,229,230^Th isotopes^39,40^, which define the well-known first island of asymmetric fission, are included. The comprehensive view provided by Fig. 2 reveals the emergence of a distinct island of asymmetric fission within the sub-lead region. This secondary island is notably evident for the most neutron-deficient lead isotopes and extends to lower *Z* elements, including platinum isotopes. Despite the asymmetric tail in the elemental yield for the bismuth isotopes (^189–197^Bi), the overall distribution exhibits clear symmetry, mirroring the pattern observed for other higher elements up to the well-established actinide region. This variability in asymmetry enabled us to experimentally define the boundaries of this island of asymmetric fission, which had previously only been delineated through theoretical calculations^31^. Notably, our experimental findings do not completely agree with the predictions of this previous theoretical study regarding the location and extent of the asymmetric island. In particular, it had been reported that the asymmetric fission island extends from ^180^Hg to ^196^Hg, and from ^183^Tl to ^195^Tl. However, our data indicate that this new asymmetric fission island does not reach systems such as ^196^Hg or ^195^Tl. For example, we observed that ^188^Tl and ^186^Hg exhibit a strong symmetric component. Furthermore, our data indicate that the asymmetric fission island extends beyond ^180^Hg and ^183^Tl towards the more neutron-deficient systems, which is not supported by the calculations in ref. ^31^. The experimental values of the asymmetry parameter ${\mathcal{A}}$ are listed in Extended Data Tables 1 and 2 along with the values obtained from GEF calculations. A comparable map based on the standard deviation of the experimental charge distributions is presented in Extended Data Fig. 6. This map, constructed using the standard deviation values provided in Extended Data Tables 1 and 2, reinforces the evidence for the emergence of the new island of asymmetric fission.

**Fig. 3:**
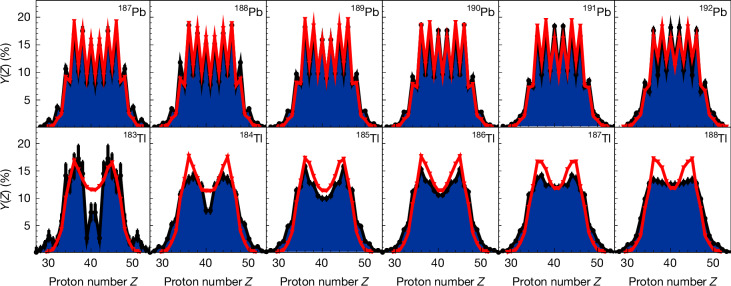
Charge yields of thallium and lead isotopes. Measured charge yields in blue are for fissioning isotopes in the second island of asymmetric fission. The red triangles represent the charge yield calculated using GEF^42^. Error bars corresponding to statistical uncertainties are visible if not smaller than the data points.

**Fig. 4:**
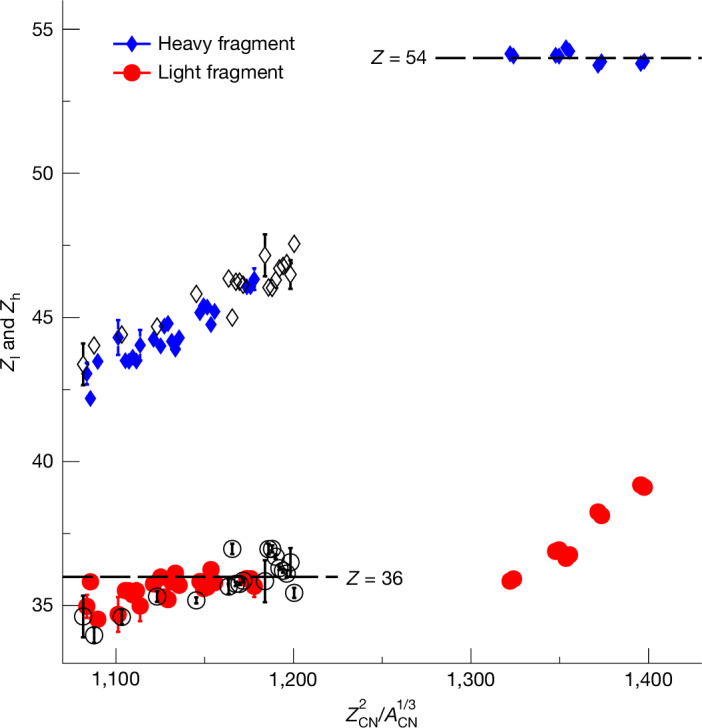
Evidence of *Z* = 36 stabilization of the light fragments. Light (red filled circle) and heavy (blue filled diamonds) charge fragments from the asymmetric fission islands as a function of the Coulomb parameter of the compound nucleus when ${{\mathcal{A}}}_{{\rm{Gauss}}} > 1$. The open symbols represent the asymmetric fission mode for symmetric fission when ${{\mathcal{A}}}_{{\rm{Gauss}}} < 1$. These have distinctly asymmetric tails. The data clearly display the well-known *Z*_h_ ≈ 54 stabilization for heavy fissioning systems as well as new *Z*_l_ ≈ 36 stabilization for pre-actinides in the new island of asymmetric fission. *Z*_CN_ and *A*_CN_ correspond, respectively, to the charge and mass of the fissioning system. When the error bars (1*σ*) do not appear, they are smaller than the data points.

Figure 3 presents the charge yields of several elements confined within the neutron-deficient sub-lead region, a pivotal domain where the onset of asymmetric fission phenomena is observed. Yields computed using the GEF code^42^ are presented in red. Because of the large number of systems studied in this experiment and the attained level of nuclear-charge resolution, the current dataset unveils systematic features of the charge distribution that could not be achieved with other experimental approaches. The data distinctly demonstrate a transition to more asymmetric fission as we move towards neutron-deficient systems. Notably, an intriguing feature emerges from the data displayed in Fig. 3: the dominant element for the charge distribution of the light fragments consistently seems to be *Z*_l_ = 36, corresponding to krypton. From platinum up to the most neutron-deficient lead isotopes, the heavier partners exhibit a range from *Z*_h_ = 42 to 46. This indicates a persistent prevalence of the lighter fragment with *Z*_l_ = 36. To isolate the primary asymmetric fission modes, we performed a global fit of the charge yields using a three-Gaussian model, as detailed in Methods. Examples of this fit are shown in Extended Data Fig. 2d–g. We defined a new asymmetry parameter ${{\mathcal{A}}}_{{\rm{Gauss}}}$ as the ratio of the asymmetric yield to the symmetric yield from the fit. When the overall distribution is asymmetric, ${{\mathcal{A}}}_{{\rm{Gauss}}}$ is greater than 1, otherwise, it is less than 1. The calculated values of ${{\mathcal{A}}}_{{\rm{Gauss}}}$ are also listed in Extended Data Tables 1 and 2. From this fit, we extracted the mean values of the different Gaussian components, especially the asymmetric contributions. These mean values correspond to the light- and heavy-fragment charge components of asymmetric fission and are plotted in Fig. 4 as filled symbols when ${{\mathcal{A}}}_{{\rm{Gauss}}} > 1$. In some cases, such as ^181^Au (Extended Data Fig. 2), the overall distribution is symmetric with ${{\mathcal{A}}}_{{\rm{Gauss}}} < 1$, yet it clearly exhibits an asymmetric contribution. For these cases, we also extracted the main *Z*_l_ and *Z*_h_ values associated with the asymmetric fission mode. These are plotted as open symbols in Fig. 4. Overall, this plot depicts the charge *Z* of both the light (red) and heavy (blue) fragments in the two islands of asymmetric fission as a function of the Coulomb parameter of the fissioning system. The global picture with the well-known *Z*_h_ ≈ 54 stabilization is substantially enriched by these new data. They clearly highlight the major role played by the deformed proton configuration at *Z* = 36 in governing the intricate dynamics of fission within the neutron-deficient sub-lead region.

Finally, the significant impact of pairing correlations on the fission behaviour of even-*Z* compound systems is prominently evident in this newly acquired dataset (Extended Data Fig. 4). Indeed, within such systems, the odd–even staggering in the fragment population appears clearly for the lead isotopes, as displayed in Fig. 3. The magnitude of this staggering is notably captured well by the GEF calculations. However, the code fails to describe properly the transition towards more symmetric fission in increasingly neutron-rich systems. This is especially visible in the thallium chain isotopes in Fig. 3. This discrepancy may indicate an incomplete understanding of the interplay between microscopic structural effects and the macroscopic potential within the current models.

**Fig. 5:**
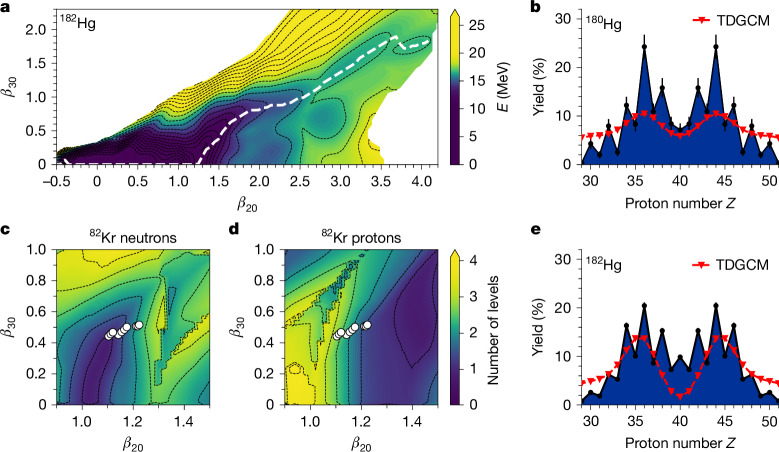
Theoretical calculations and comparison with experimental data. **a**, Two-dimensional PES of ^182^Hg as a function of its quadrupole and octupole moments. **b**,**e**, Experimental charge yields with statistical uncertainties as error bars, compared to those obtained from the TDGCM calculations for ^180^Hg (**b**) and ^182^Hg (**e**). **c**,**d**, Smoothed number of levels around the neutron (**c**) and proton (**d**) Fermi levels^46^ for the favoured light pre-fragment ^82^Kr from ^182^Hg fission. A low number of levels indicates stabilization by a shell effect. The white circles represent the deformation of ^82^Kr when approaching the scission line of ^182^Hg.

State-of-the-art microscopic calculations were performed in the mercury region for even–even fissioning nuclei, using the self-consistent constrained Hartree–Fock–Bogoliubov approximation with the Gogny D1S functional. By applying constraints on the quadrupole (*β*_20_) and octupole (*β*_30_) moment operators, we generated the two-dimensional potential energy surfaces (PESs) describing the static deformation properties of these nuclei. Similar calculations constraining only the quadrupole moment operator yielded the one-dimensional adiabatic path of the fission reaction. Figure 5a depicts the static properties for ^182^Hg fission. The one-dimensional reaction path follows an asymmetric valley of the potential, leading mostly to configurations with a ^82^Kr (*Z* = 36) light pre-fragment. The shell effects have been systematically studied along the one-dimensional reaction path by determining the level density close to the Fermi level following the description given in refs. ^46,47^. This revealed that the potential valley present at elongations close to *β*_20_ = 3.9 is, indeed, correlated to shell effects in the fissioning system as a whole as well as in its light pre-fragment. Figure 5c,d illustrates the latter. The ^82^Kr deformations found on approaching the scission of ^182^Hg are represented by white circles. We show here that these deformations lie within the area of both quadrupole–octupole deformed neutron (*β*_20_ ≈ 1.1 and *β*_30_ *≈* 0.5) and proton (*β*_20_ ≈ 1.4 and *β*_30_ *≈* 0.5) shells, confirming a proton shell at *Z*_l_ = 36, which is accompanied by a neutron shell at *N*_l_ = 46. A systematic application of this method to the fission of even–even platinum, mercury and lead isotopes leads to the conclusion that the shell effects responsible for the asymmetric split are in the light fragments, in contrast to the actinide region, where the asymmetric fission trajectory is predominantly influenced by the heavy fragment. Systematic details and specific examples are given in Methods, and a summary is given in Table 1. We found that the strong quadrupole–octupole deformed *Z* = 36 shell shown in Fig. 5d also appears for ^80^Kr and is the main driver of asymmetric fission in this region.

**Table 1:** Compound nuclei for which a krypton isotope appears as a driver on the way to asymmetric fission

Compound	*Z*_l_	*N*_l_	*Z*_h_	*N*_h_
^174^Pt	**36**	44	42	**52**
^176^Pt	**36**	**46**, 44	42	52, 50
^178^Pt	**36**	**46**	42	54
^180^Hg	**36**	**46**	44	54
^182^Hg	**36**	44	44	58
^190^Pb	**36**	**46**	46	62
^192^Pb	36	44	46	**66**
^194^Pb	36	**50**	**46**	**62**

Corresponding quadrupole–octupole deformed shells are represented by bold numbers.

To complete this interpretation based solely on the static deformation properties of the nuclei, we simulated the fission dynamics in the neutron-deficient mercury region using the time-dependent generator coordinate method (TDGCM) under the Gaussian overlap approximation^48^. Figure 5b,e presents the resulting charge yields for the fission of ^180^Hg and ^182^Hg. The dynamical calculations produced a *Z*_l_ ≃ 36 peak, consistent with experimental data. We also found that the difference of two neutrons between ^180^Hg and ^182^Hg modifies the most probable heavy pre-fragment and leaves the number of neutrons in the light pre-fragment invariant. This is further evidence for a fission process driven by a shell effect in the light fragment. A robust conclusion from the dynamics on the whole mercury chain is, at this point, still elusive. This is due to the inability to generate continuous PESs from the ground state up to scission for such highly neutron-deficient fissioning systems. It is probable that this limitation in the description is also responsible for the missing odd and even staggering in the predicted charge distributions, as discussed in ref. ^49^. Several promising new theoretical approaches may overcome this difficulty in the coming years^50–52^, and our data will continue to serve as a critical benchmark of these future calculations.

## Conclusion

In summary, we provide a set of charge distributions across a wide range of exotic fissioning systems, from neutron-deficient iridium to thorium isotopes. Fission was induced at excitation energies of 5 to 10 MeV above the fission barrier. The precision achieved in charge resolution, facilitated by our experimental method with inverse kinematics at relativistic energies, has yielded compelling evidence of proton-shell stabilization at *Z* = 36 within the light fission fragment in an island of asymmetric fission. Unlike actinides, the asymmetric fission of this island is driven by the light fragment. Using this extensive dataset, we mapped this new island of asymmetric fission and we also established constraints for fission models, testing their accuracy and predictive capability in calculating fission fragment distributions for systems with an extreme neutron-to-proton ratio. This discovery significantly impacts our understanding of fragment distribution within the island of asymmetric fission and will challenge existing and future theoretical investigations. To complete this dataset, future experiments will extend the study to even more neutron-deficient systems, aiming to investigate further the western boundaries of the asymmetric fission island.

## Methods

### Beam production

**Extended Data Fig. 1:**
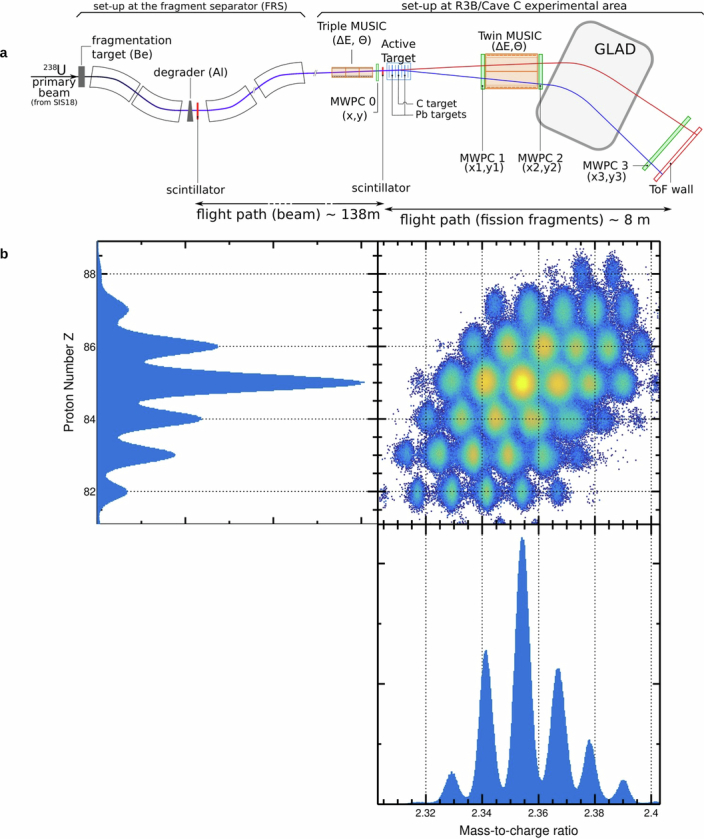
Schematic view of the experimental setup and particle identification plots. **a**, Schematic view of the FRagment Separator (FRS) together with the experimental apparatus at R^3^B (figure generated using Inkscape on Linux). **b**, Beam identification plot for FRS settings including ^200^At in the cocktail beam. The projections give the charge *Z* and mass-to-charge ratio *A*/*Q* resolution for the charge *Z* = 85.

The experiment was performed at GSI in Germany within the R^3^B-SOFIA collaboration. A ^238^U primary beam was accelerated with the SIS18 synchrotron up to 1 GeV per nucleon. The primary beam impinged on a 1,625 mg cm^−^^2^ thick beryllium target at the entrance of the FRS. Nb stripper foil 220 mg cm^−^^2^ thick was attached to the exit side of the target to produce a high fraction of fully ionized fragments. The secondary beams, produced by fragmentation, were selected with *Bρ*–Δ*E*–*Bρ* by the FRS and transported to the experimental area. The different incoming beams were identified event by event using the Δ*E*–*Bρ*–ToF method. Indeed, the atomic number *Z* of the incoming ion was obtained from the energy loss Δ*E* deposited in an ionization chamber^53^ placed at the entrance of the experimental area. The mass number *A* was determined from its magnetic rigidity *A*/*Q* = *Bρ*/*βγ*, with *β* = *v*/*c* the velocity of the ion and *γ* the Lorentz factor. In the magnetic rigidity *Bρ*, *B* is the magnetic field and *ρ* is the radius of curvature of the particle’s trajectory in the magnetic field. The magnetic rigidity was obtained from a horizontal position measurement *x* at the dispersive focal plane of the FRS associated with the central magnetic rigidity *Bρ*_0_ of the setting. The bottom of Extended Data Fig. 1 displays the beam identification for those FRS settings that include ^200^At in the cocktail beam. All nuclei of interest were well identified and separated with resolutions of Δ*Z*/*Z* = 2.5 × 10^−3^ and Δ*A*/*A* = 7.7 × 10^−4^, as one can see from the respective projections in Extended Data Fig. 1.

### Experimental set-up

Extended Data Fig. 1 shows the global scheme of the experimental set-up. This figure was generated using Inkspace on Linux.

### Secondary fission reactions

Fission was induced in the active target, which was a detector dedicated to determining the cathode in which the fission took place. The target consisted of a cylindrical volume filled with P10 gas. It had two 1.5-mm-thick lead cathodes and one 0.5-mm-thick carbon cathode. High-*Z* targets were used because they enhanced the Coulomb interaction without significantly increasing the nuclear contribution. The carbon target was then used to account for fission induced by nuclear interactions. Locating where the fission occurred was obtained from measuring the energy loss between one cathode and one anode. If fission did not occur, then the initial ion passed the volume with a typical energy loss proportional to the square of its charge $\Delta E\propto {Z}_{{\rm{CN}}}^{2}$, where *Z*_CN_ is the charge of the compound fissioning nuclei. If fission did happen, then the energy loss corresponded to the sum of the energies deposited by the two fragments $\Delta E\propto {Z}_{1}^{2}+{Z}_{2}^{2}\approx {Z}_{{\rm{CN}}}^{2}/2$. As a consequence, correlating the energy between the different successive anodes allowed us to determine the cathode in which fission took place.

**Extended Data Fig. 2:**
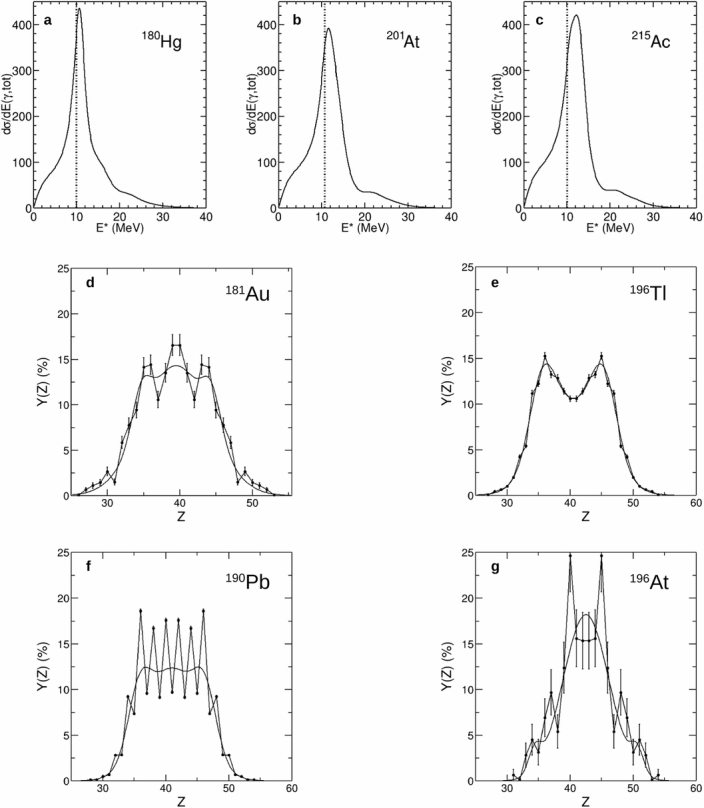
Excitation energy distribution and example of Gaussian fit of the charge yields. **a**–**c**, Example of three excitation energy distributions for lower-, middle- and higher *Z* values. The dashed lines correspond to the fission barrier listed in Extended Data Tables 3 and 4. **d**–**g**, Three-Gaussian fit of the charge yields for a selection of fissioning systems.

The excitation energy distribution of the projectile following Coulomb excitation was calculated for all systems studied using the empirical parameterization of giant resonances^41^ and the ground-state deformation data from the AMEDEE database^43^. Extended Data Fig. 2a–c illustrates this distribution for three fissioning systems. The calculated fission barriers are depicted as dashed lines. In the GEF code, the fission barrier is calculated as the sum of the macroscopic fission barrier and the ground-state shell-correction energy using the topographic theorem^42^. The macroscopic component of the fission barrier corresponds to the Thomas–Fermi barriers.

### Identifying fission fragments

**Extended Data Fig. 3:**
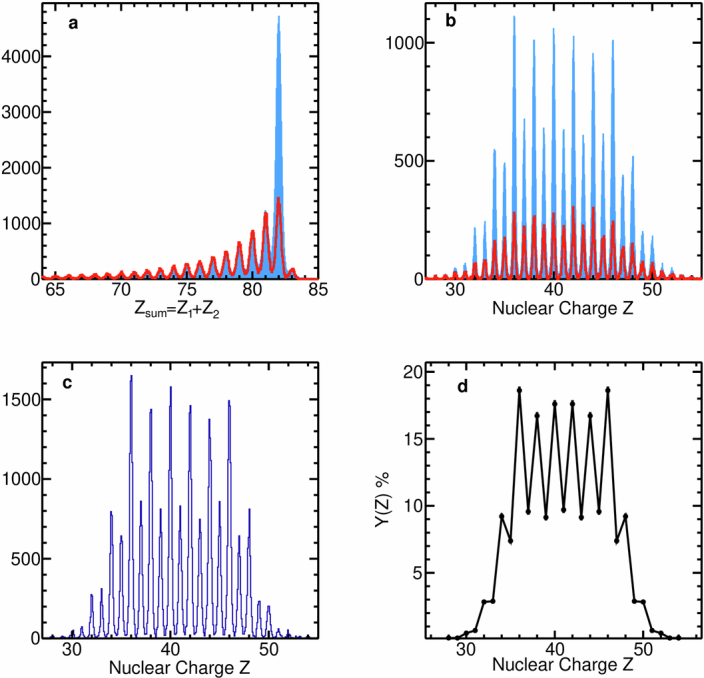
Experimental measured charge distribution and associated yield. **a**, Sum of both fragment charge *Z*_∑_ = *Z*_1_ + *Z*_2_ in blue when the fission is induced in the lead targets and in red when it is induced in the carbon target. **b**, Atomic charge distribution after fission of ^190^Pb induced in the lead cathodes (blue) and the carbon cathode (red) with the condition *Z*_∑_ = 82. **c**, Atomic charge distribution from the electromagnetic induced fission where the fragmentation/fission contribution has been subtracted with the condition *Z*_∑_ = 82. **d**, Final charge yield for the Coulomb-induced fission of ^190^Pb. The error bars are depicted.

**Extended Data Fig. 4:**
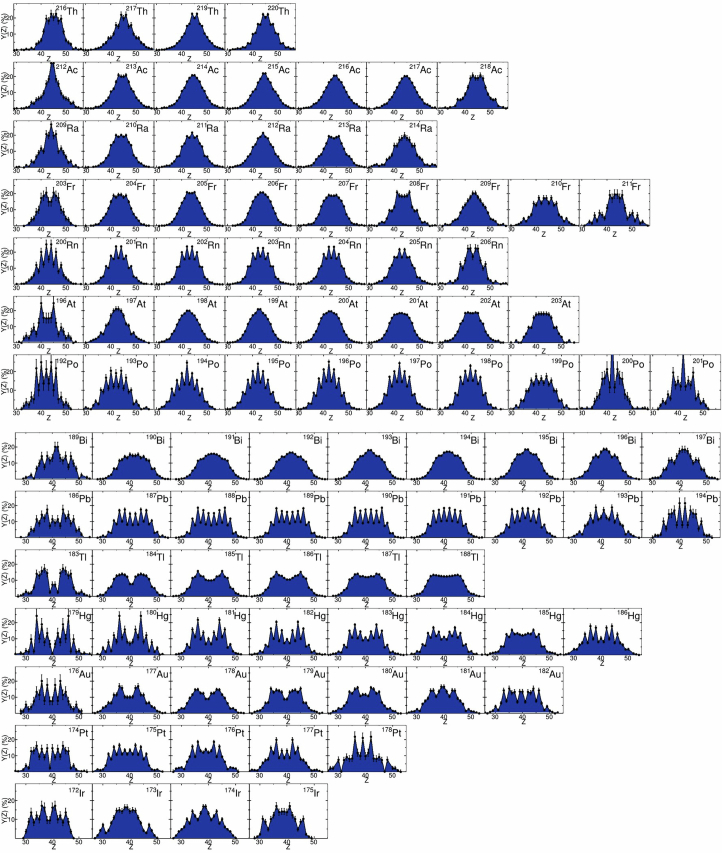
Measured charge yields. Experimental measured charge yields from iridium to thorium isotopes.

In this analysis, we focused only on events where the sum of the charges ∑*Z *of both fission fragments was equal to the charge of the fissioning system. However, a remaining component of the nuclear excitation was still present for fragmentation if a proton was not emitted before fission. Those events were subtracted using data from the low-*Z* carbon cathode. The atomic numbers *Z* of both fission fragments were measured in a dedicated ionization chamber, Twin-MUSIC (multi-sample ionization chamber). This detector was segmented into four sections to maximize the detection efficiency for both fragments. The loss of efficiency was mainly due to fragments passing through the cathode of the MUSIC detector. The measured energy deposited in this ionization chamber required correction before we could infer the atomic numbers of the fission fragments. In particular, due to the wide velocity distribution of the fission fragments, the energy loss was corrected for the *β* dependence. Extended Data Fig. 3b displays in blue the charge distribution from the fission induced in the lead cathodes when the sum of the charges of both fragments was equal to the charge of the fissioning system (∑Z= 82 here). The spectrum in red shows the charge distribution with the same conditions but from the carbon cathode.

Extended Data Fig. 3c shows the final charge distribution from electromagnetically induced fission after the nuclear contribution has been subtracted. An average resolution of *σ* = 0.13 charge units was obtained for all fission fragments. The final charge yields of the fission fragments, normalized to 200%, are displayed in Extended Data Fig. 3d. This work was done systematically all the fissioning systems studied.

### Gaussian fitting of the charge yields

All distributions were systematically fitted using three Gaussian functions. The first and third Gaussians, corresponding to the asymmetric components of the distribution, had the same height and sigma parameters. All other parameters were allowed to vary freely. The symmetric Gaussian, however, had three free parameters.

Extended Data Fig. 2d–g shows examples of the fitting. The fits for ^181^Au and ^186^Tl reproduce the distributions very well. By contrast, fitting ^190^Pb was more challenging due to the pronounced odd–even staggering in its distribution. Although the statistics are limited for ^196^At, the fit clearly identifies a symmetric component in the distribution.

Using this three-Gaussian fit, we defined a new asymmetry variable ${{\mathcal{A}}}_{{\rm{Gauss}}}$ as the ratio of the asymmetric yield to the symmetric yield from the fit. When the overall distribution was asymmetric, ${{\mathcal{A}}}_{{\rm{Gauss}}}$ was greater than 1, otherwise, it was less than 1. Calculated values of ${{\mathcal{A}}}_{{\rm{Gauss}}}$ are listed in Extended Data Tables 1 and 2.

From this fit, we extracted the mean values of the different Gaussian components, particularly for the asymmetric contributions. These mean values correspond to the light-fragment charge and the heavy-fragment charge components of asymmetric fission. In some cases, such as ^181^Au (Extended Data Fig. 2), the overall distribution was symmetric with ${{\mathcal{A}}}_{{\rm{Gauss}}} < 1$, yet it clearly exhibits an asymmetric contribution. For these cases, we also extracted the main *Z*_l_ and *Z*_h_ values associated with the asymmetric fission mode.

### Data acquisition and trigger condition

GSI data were acquired for the experiment. Only multiplicity 2 in the Tof-wall detector triggered the acquisition. Thus, only events with two fragments hitting the Tof-wall in two different plastic paddles resulted in a valid trigger signal.

### Shell effects within the one-dimensional asymmetric path before scission

Given the consistent emergence of *Z* = 36 fragments in our experimental data, our study, which was based on mean-field calculations, was extended to investigate the role of krypton isotopes in the PES and encompass stages close to scission. This exploration revealed the ubiquitous presence of krypton isotopes in the asymmetric fission pathways of the Pt, Hg and Pb chains, thus emphasizing their leading role. A selection of 12 even–even systems were considered. In nine of them, krypton isotopes appeared experimentally as primary light fragments. When doing the theoretical analysis of the shell effect described for this set of nuclei, we separated a compound nucleus into two pre-fragments along the asymmetric path before scission. We found that krypton isotopes appeared in eight systems on the way to scission along a typical distance of *β*_2_ ≈ 0.6 within the compound-nucleus PES, further clarifying the enduring influence of the deformed *Z* = 36 shell in steering asymmetric fission within the lead region. This is summarized in Table 1, which also gives other pre-fragment particle numbers. Even if the final configuration before scission does not provide a *Z* = 36 light pre-fragment, the compound nuclei presented in Table 1 had a *Z* = 36 light pre-fragment for a significant distance along the final stage of the asymmetric path, with is a typical *β*_2_ ≈ 0.6 trajectory within a compound-nucleus PES. When the system experienced a pre-fragment shell effect along the asymmetric path before scission, the particle number is denoted in bold. Most shell effects were found for the krypton light pre-fragment, and most were for *Z* = 36. To a lesser extent, the effect of the deformed *N* = 46 shell was also predominant. This is the case, for example, for ^182^Hg (Fig. 5c). Shell effects around the Fermi level were detected with the smoothed level density (SLD) tool defined in ref. ^46^. Low SLD regions in the (*β*_2_, *β*_3_) planes define the localization of strong local shell effects. In Fig. 5c, most of the trajectory occurred within a *N* = 46 low SLD zone, which emphasizes the role of *N* = 46 in this specific situation. Nevertheless, a deeper analysis of these three chains showed that the *N* = 46 shell appeared only for the krypton isotope. Thus, the *Z* = 36 and *N* = 46 shell make ^82^Kr a strong driver of asymmetric fission in this region. In the following, we consider ^174^Pt and ^190^Pb. Both illustrate the influence of the strong effect of the proton *Z* = 36 shell in ^80^Kr and ^82^Kr, respectively.

#### ^174^Pt

**Extended Data Fig. 5:**
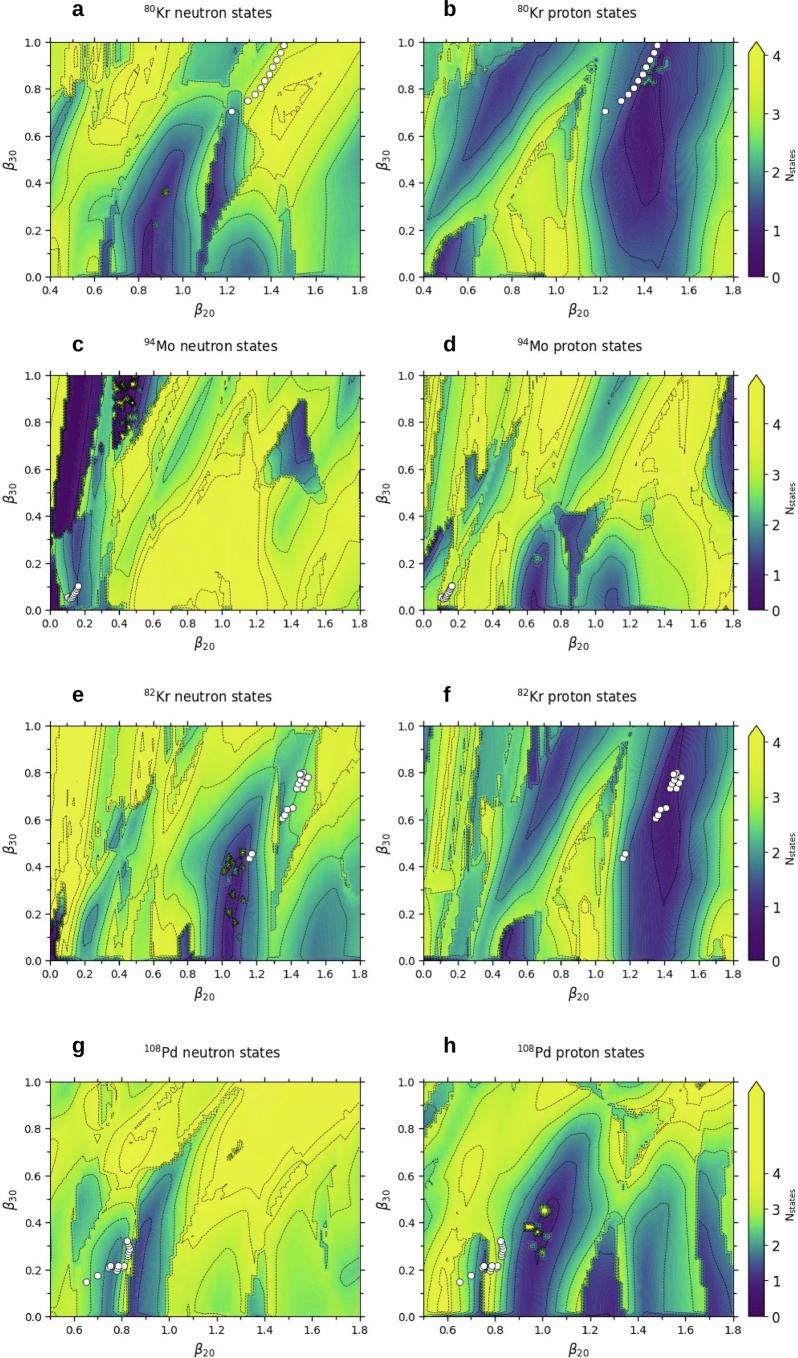
Calculated shell effects the the fragments. ^80^Kr shell effects at the Fermi level for neutrons (**a**) and protons (**b**). The white dots trace the light prefragment of ^174^Pt before scission. ^94^Mo shell effects at the Fermi level for neutrons (**c**) and protons (**d**). The white dots trace the heavy prefragment of ^174^Pt before scission. ^82^Kr shell effects at the Fermi level for neutrons (**e**) and protons (**f**). The white dots trace the light prefragment of ^190^Pb before scission. ^108^Pd shell effects at the Fermi level for neutrons (**g**) and protons (**h**). The white dots trace the heavy prefragment of ^190^Pb before scission.

**Extended Data Fig. 6:**
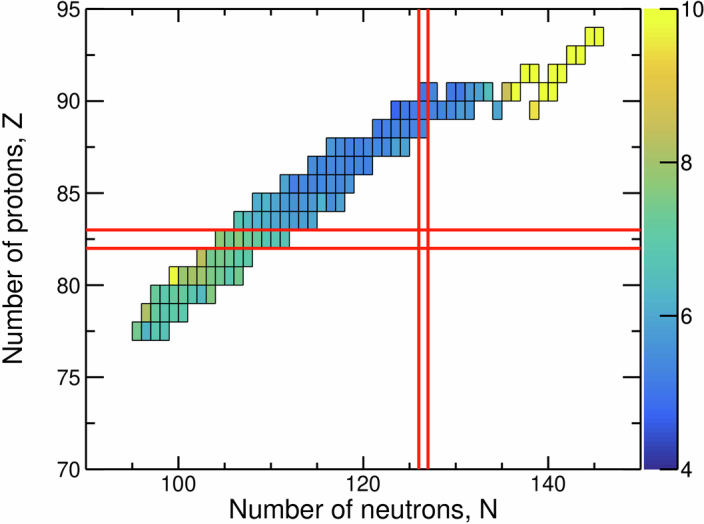
Asymmetric fission map using RMS of the charge distribution. Experimental asymmetry using the RMS of the charge distribution as metric listed in Extended Data Tables 1 and 2.

Manifestations of the *Z* = 36 shell are depicted in Extended Data Fig. 5a,b through the ^80^Kr isotope. When approaching scission along the asymmetric path of ^174^Pt, the separation into pre-fragments from the density minimum gave rise to a ^80^Kr light pre-fragment and a ^94^Mo heavy one (Extended Data Fig. 5c,d). Along this trajectory, ^80^Kr was found in a low SLD region caused by a strong proton shell (as seen in Extended Data Fig. 5b), like that found for ^82^Kr. This shell was centred around *β*_2_ ≈ 1.4 and ranged from *β*_3_ = 0 up to *β*_3_ > 1. The strongest region in this low SLD was localized around *β*_3_ ≈ 0.5, which gives ^80^Kr the typical pear-shaped deformation seen for fragments at scission. On the heavy pre-fragment side, a neutron *N* = 52 shell was also found, as noted in bold in Table 1 and shown in Extended Data Fig. 5c. The evolution of the whole compound deformation was such that both pre-fragments remained within their own shell effect. As the *Z* = 36 shell is much wider than the *N* = 52 shell, ^80^Kr absorbed most of the deformation. For the three nuclei involving ^80^Kr listed in Table 1, the same quadrupole and octupole deformation at proton number *Z* = 36 characterized by a low smoothed level density appears, emphasizing the deformed nature of this shell.

#### ^190^Pb

Another insight into the role of the *Z* = 36 shell is depicted in Extended Data Fig. 5e–h for ^190^Pb. The SLDs plotted against their quadrupole and octupole deformations are depicted in Extended Data Fig. 5e,f for the light ^82^Kr pre-fragment. Their respective deformations within the ^190^Pb path are shown by trajectories within the SLD plots. During the last stages of ^190^Pb fission, ^82^Kr remained in a low *Z* = 36 SLD (left panel), the deformation of ^82^Kr being stabilized. By contrast, the ^108^Pd trajectory (Extended Data Fig. 5g,h) did not reveal any real strong correlations with the proton or neutron shell effect (only a small region, characterized by a low neutron and proton SLD, is traversed during the trajectory). Thus, the deformed *Z* = 36 shell is the main driver of asymmetric ^190^Pb fission.

More generally, ^82^Kr was found as pre-fragments for various compound nuclei as presented in Table 1. Extended Data Fig. 5e,f presents two strongly deformed shells: a neutron one around *β*_2_ ≈ 1.1 and a substantial proton one around *β*_2_ ≈ 1.4. These shells also have a strong octupole deformed character, ranging from *β*_3_ = 0 to *β*_3_ ≈ 0.6 and *β*_3_ > 1 on the neutron and proton sides, respectively. The strongest region in the *Z* = 36 low SLD is especially localized around *β*_3_ ≈ 0.5, which gives ^82^Kr the same typical pear-shaped deformation expected for fragments at scission, as found for ^82^Kr in the first example.

These results contrast with the actinide region, where the asymmetric fission trajectory is predominantly driven by the heavy fragment. One might ask why *Z* = 36 is not known for having a strong shell effect in the actinide region. A first part of the answer is that compact asymmetric fission modes are dominant in this region. Thus, the compound nucleus scissions before reaching the deformations needed to reach the krypton shell. In addition, the krypton isotopes one might expect as actinide fragments are very exotic neutron-rich nuclei, far from those in the lead region that have strong shells (^80–82^Kr).

## Online content

Any methods, additional references, Nature Portfolio reporting summaries, source data, extended data, supplementary information, acknowledgements, peer review information; details of author contributions and competing interests; and statements of data and code availability are available at 10.1038/s41586-025-08882-7.

## Figures and Tables

**Extended Data Table 1:**
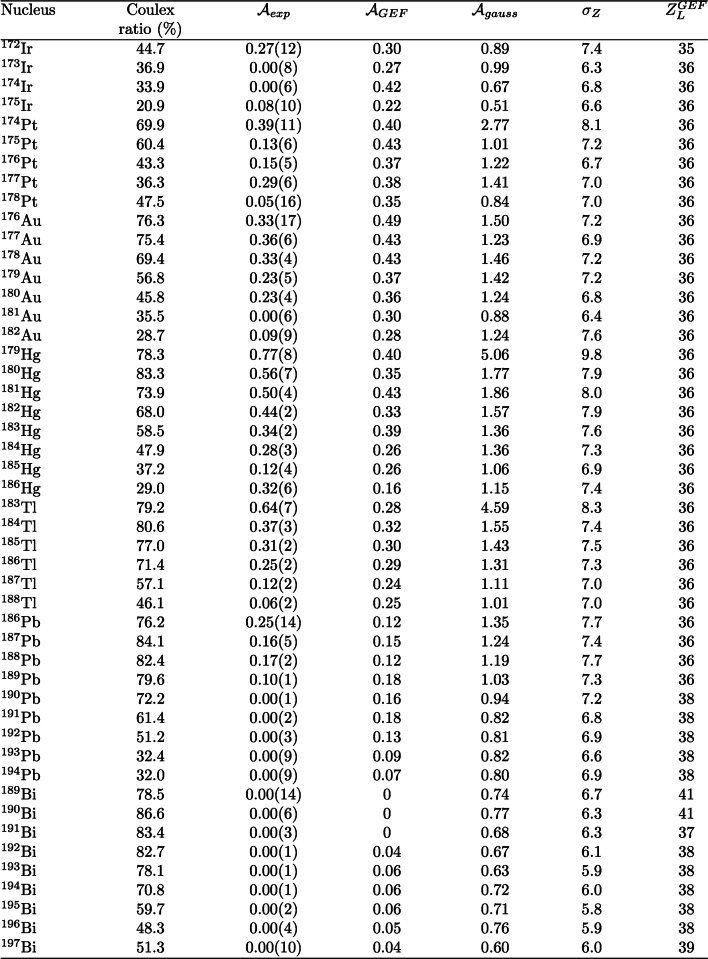
List of studied nuclei from Ir to Bi isotopes, with the percentage of Coulomb-induced fission events, the asymmetry parameter value from equation (1) for the experimental data ${\boldsymbol{\mathcal{A}}}_{{\boldsymbol{e}}{\boldsymbol{x}}{\boldsymbol{p}}}$ and for GEF ${\boldsymbol{\mathcal{A}}}_{{\boldsymbol{GEF}}}$, the asymmetry parameter value from the Gaussian-fit method ${\boldsymbol{\mathcal{A}}}_{{\boldsymbol{gauss}}}$, the standard deviation *σ*_*Z*_ of the experimental charge distribution, and the light charge corresponding to the highest yield from GEF calculation ${{\boldsymbol{Z}}}_{{\boldsymbol{L}}}^{{\boldsymbol{GEF}}}$

**Extended Data Table 2:**
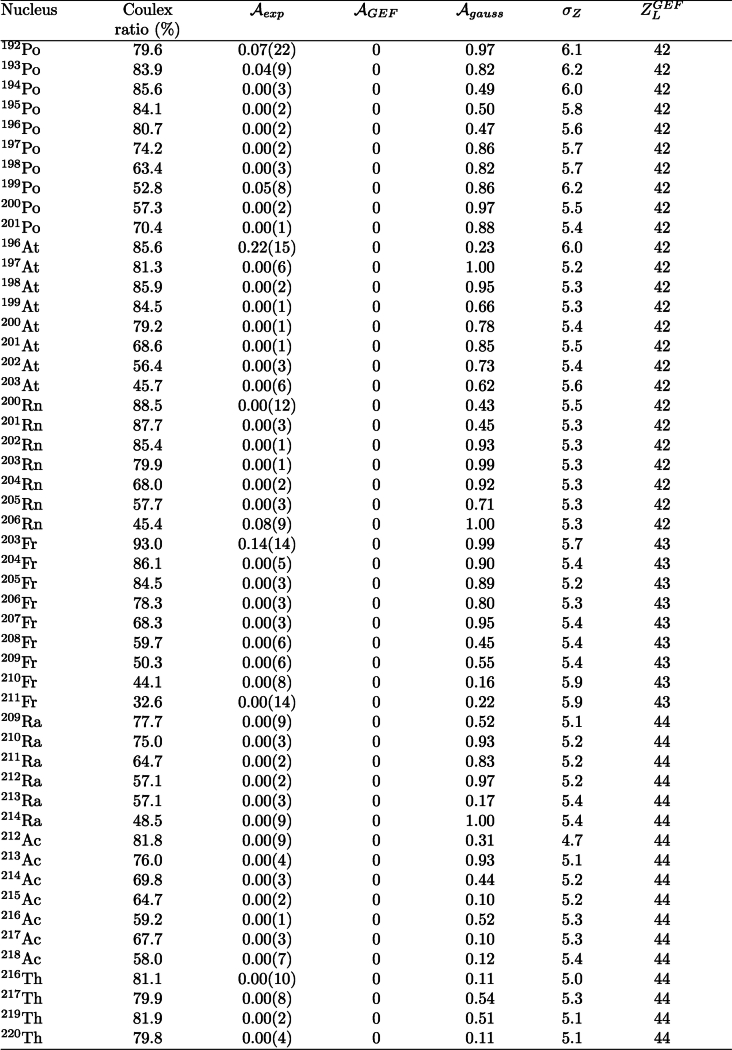
List of studied nuclei from Po to Th isotopes, with the percentage of Coulomb-induced fission events, the asymmetry parameter value from equation (1) for the experimental data ${\boldsymbol{\mathcal{A}}}_{{\boldsymbol{e}}{\boldsymbol{x}}{\boldsymbol{p}}}$ and for GEF ${\boldsymbol{\mathcal{A}}}_{{\boldsymbol{GEF}}}$, the asymmetry parameter value from the Gaussian-fit method ${\boldsymbol{\mathcal{A}}}_{{\boldsymbol{gauss}}}$, the standard deviation *σ*_*Z*_ of the experimental charge distribution, and the light charge corresponding to the highest yield from GEF calculation ${{\boldsymbol{Z}}}_{{\boldsymbol{L}}}^{{\boldsymbol{GEF}}}$

**Extended Data Table 3:**
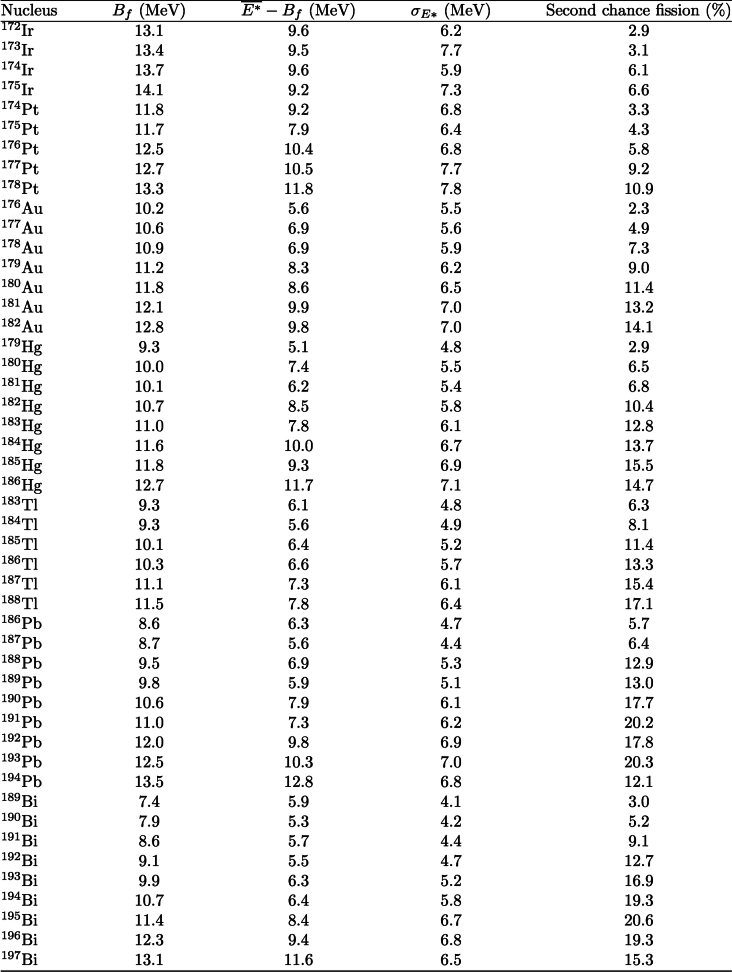
List of studied nuclei from Ir to Bi isotopes, with the fission barrier estimated by using the GEF code together with the average mean excitation energy of the fissioning system above the fission barrier, the standard deviation of the excitation energy distribution, and the percentage of second chance fission

**Extended Data Table 4:**
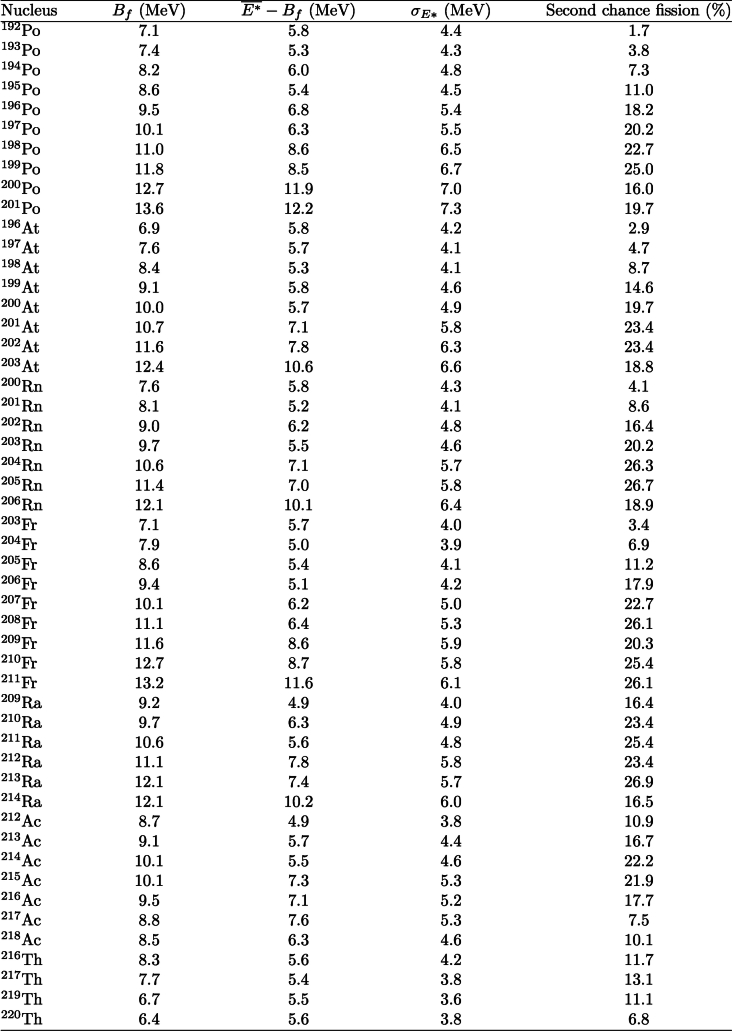
List of studied nuclei from Po to Th isotopes, with the fission barrier estimated by using the GEF code together with the average mean excitation energy of the fissioning system above the fission barrier, the standard deviation of the excitation energy distribution, and the percentage of second chance fission

## Data Availability

The data used in this study originate from the s455 GSI experiment. The raw data are available on request. The source data for Fig. 2, which displays the asymmetric parameter, are provided in Extended Data Tables 1 and 2.

## Code availability

The main analysis and results presented in this paper were performed using the nptool framework. The analysis codes used to generate these results are available in the s455 project of nptool at https://gitlab.in2p3.fr/np/nptool/-/tree/NPTool.2.dev/Projects/s455?ref_type=heads.
