# Author Correction: An asymmetric fission island driven by shell effects in light fragments

**DOI:** 10.1038/s41586-025-09936-6

**Published:** 2025-11-25

**Authors:** P. Morfouace, J. Taieb, A. Chatillon, L. Audouin, G. Blanchon, R. N. Bernard, N. Dubray, N. Pillet, D. Regnier, H. Alvarez-Pol, F. Amjad, P. André, G. Authelet, L. Atar, T. Aumann, J. Benlliure, K. Boretzky, L. Bott, T. Brecelj, C. Caesar, P. Carpentier, E. Casarejos, J. Cederkäll, A. Corsi, D. Cortina-Gil, A. Cvetinović, E. De Filippo, T. Dickel, M. Feijoo, L. M. Fonseca, D. Galaviz, G. García-Jiménez, I. Gasparic, E. I. Geraci, R. Gernhäuser, B. Gnoffo, K. Göbel, A. Graña-González, E. Haettner, A.-L. Hartig, M. Heil, A. Heinz, T. Hensel, M. Holl, C. Hornung, A. Horvat, A. Jedele, D. Jelavic Malenica, T. Jenegger, L. Ji, H. T. Johansson, B. Jonson, B. Jurado, N. Kalantar-Nayestanaki, E. Kazantseva, A. Kelic-Heil, O. A. Kiselev, P. Klenze, R. Knöbel, D. Körper, D. Kostyleva, T. Kröll, N. Kuzminchuk, B. Laurent, I. Lihtar, Yu. A. Litvinov, B. Löher, N. S. Martorana, B. Mauss, S. Murillo Morales, D. Mücher, I. Mukha, E. Nacher, A. Obertelli, E. V. Pagano, V. Panin, J. Park, S. Paschalis, M. Petri, S. Pietri, S. Pirrone, G. Politi, L. Ponnath, A. Revel, H.-B. Rhee, J. L. Rodríguez-Sánchez, L. Rose, D. Rossi, P. Roy, P. Russotto, C. Scheidenberger, H. Scheit, H. Simon, S. Storck-Dutine, A. Stott, Y. L. Sun, C. Sürder, Y. K. Tanaka, R. Taniuchi, O. Tengblad, I. Tisma, H. T. Törnqvist, M. Trimarchi, S. Velardita, J. Vesic, B. Voss, F. Wamers, H. Weick, F. Wienholtz, J. Zhao, M. Zhukov

**Affiliations:** 1 CEA, DAM, DIF, Arpajon, France; 2 Université Paris-Saclay, CEA, Laboratoire Matière en Conditions Extrêmes, Bruyères-le-Châtel, France; 3 Université Paris-Saclay, CNRS, IJC Lab, Orsay, France; 4 CEA, DES, IRESNE, DER, SPRC, Cadarache, Saint-Paul-Lès-Durance, France; 5 IGFAE, University of Santiago de Compostela, Santiago de Compostela, Spain; 6 GSI Helmholtzzentrum für Schwerionenforschung, Darmstadt, Germany; 7 CEA Saclay, IRFU/DPhN, Centre de Saclay, Gif-sur-Yvette, France; 8 Technische Universität Darmstadt, Fachbereich Physik, Institut für Kernphysik, Darmstadt, Germany; 9 Helmholtz Research Academy Hesse for FAIR (HFHF), GSI Helmholtzzentrum für Schwerionenforschung, Darmstadt, Germany; 10 Instituto de Física Corpuscular, CSIC - Univ. of Valencia, Valencia, Spain; 11 Goethe-Universität Frankfurt, Frankfurt am Main, Germany; 12 Jozef Stefan Institute, Ljubljana, Slovenia; 13 CINTECX, Universidade de Vigo, DSN, Department of Mechanical Engineering, Vigo, Spain; 14 Department of Physics, Lund University, Lund, Sweden; 15 INFN Sezione di Catania, Catania, Italy; 16 II. Physikalisches Institut, Justus-Liebig-Universität, Giessen, Germany; 17 Instituto de Física, Universidade Federal Fluminense, Niterói, Brazil; 18 Laboratório de Instrumentação e Física Experimental de Partículas, LIP, Lisbon, Portugal; 19 Physics Department, Faculty of Sciences, University of Lisbon, Lisbon, Portugal; 20 RBI Zagreb, Zagreb, Croatia; 21 Dipartimento di Fisica e Astronomia ‘Ettore Majorana’, Università degli Studi di Catania, Catania, Italy; 22 Technische Universität München, Garching, Germany; 23 Institutionen för Fysik, Chalmers Tekniska Högskola, Gothenburg, Sweden; 24 Helmholtz-Zentrum Dresden-Rossendorf, Institute of Radiation Physics, Dresden, Germany; 25 Université de Bordeaux, CNRS, LP2IB, Gradignan, France; 26 ESRIG, University of Groningen, Groningen, The Netherlands; 27 School of Physics, Engineering and Technology, University of York, York, UK; 28 University of Guelph, Guelph, Ontario Canada; 29 Institut für Kernphysik der Universität zu Köln, Cologne, Germany; 30 INFN Laboratori Nazionali del Sud, Catania, Italy; 31 Institute for Basic Science, Center for Exotic Nuclear Studies, Daejeon, Republic of Korea; 32 CITENI, Campus Industrial de Ferrol, Universidade de Coruña, Ferrol, Spain; 33 Instituto de Estructura de la Materia, CSIC, Madrid, Spain; 34 Università degli Studi di Messina, Messina, Italy

**Keywords:** Experimental nuclear physics, Nuclear fusion and fission, Nuclear astrophysics

Correction to: *Nature* 10.1038/s41586-025-08882-7 Published online 30 April 2025

This article was originally published under standard Springer Nature license (© The Author(s), under exclusive licence to Springer Nature Limited). It is now available as an open-access paper under a Creative Commons Attribution 4.0 International license, © The Author(s). The error has been corrected in the HTML and PDF versions of the article.

